# Role of SALL4 in the progression and metastasis of colorectal cancer

**DOI:** 10.1186/1423-0127-20-6

**Published:** 2013-01-30

**Authors:** Mohammad Mahdi Forghanifard, Meysam Moghbeli, Reza Raeisossadati, Alireza Tavassoli, Afsaneh Javdani Mallak, Samaneh Boroumand-Noughabi, Mohammad Reza Abbaszadegan

**Affiliations:** 1 Department of Biology, Damghan Branch, Islamic Azad University, Cheshmeh-Ali Boulevard, Sa’dei Square, P.O.Box: 3671639998, Damghan, Iran; 2 Division of Human Genetics, Immunology Research Center, Avicenna Research Institute, Mashhad University of Medical Sciences, Mashhad, Iran; 3 Endoscopic and Minimally Invasive Research Center, Qaem Hospital, Mashhad, Iran; 4 Young Researchers Club and Elites, Mashhad Branch, Islamic Azad University, Mashhad, Iran; 5 Department of Pathology, Imam Reza Hospital, Mashhad University of Medical Sciences, Mashhad, Iran; 6 Department of Pathology, Ghaem Hospital, Mashhad University of Medical Sciences, Mashhad, Iran; 7 Medical Genetics Research Center, Medical School, Mashhad University of Medical Sciences, Mashhad, Iran

**Keywords:** Colorectal cancer, SALL4, Expressional analysis, Real-time PCR, Self-renewal

## Abstract

**Background:**

Human cancer cells resemble stem cells in expression signatures leading them to share some features, most notably, self-renewal. A complex network of transcription factors and signaling molecules are required for continuance of this trait. SALL4 is a zinc finger transcriptional activator crucial for maintenance of self-renewal in stem cells; however, its expression level has not yet been elucidated in colorectal tumor cells. To determine this level and probable clinicopathological consequences, its expression was analyzed.

**Methods:**

SALL4 expression in fresh tumoral and distant tumor-free tissues from 46 colorectal samples was compared by real-time polymerase chain reaction (PCR).

**Results:**

Greater than a two-fold increase in SALL4 expression was detected in 87% of tumors vs. normal related tissues. SALL4 expression was significantly correlated with tumor cell metastasis to lymph nodes, especially in moderately-differentiated tumor samples (P < 0.05). Furthermore, higher levels of SALL4 mRNA expression were significantly associated with younger than older patients with tumor cells in stages I and II (P < 0.05).

**Conclusions:**

These results indicate a relationship between SALL4 expression and tumor cell metastasis to lymph nodes and consequent advancement of tumors to advanced stages III and IV. Along with the promising evidence of its role in self-renewal in various cancers, SALL4 may have a role in progression, development and maintenance of colorectal cancers.

## Background

Colorectal cancer (CRC) is the third most common cancer in men and the second in women worldwide [1]. Almost 60% of the cases occur in developed regions where the age standardized rate for incidence (ASR) is 37.6/10^5^ in males and 24.2/10^5^ in females. Causing approximately 8% of all cancer deaths, CRC is the fourth most common cause of cancer-related death globally. The ASR for mortality in developed regions is 15.1/10^5^ and 9.7/10^5^ in males and females, respectively [1]. This cancer develops in a multistep progression; from normal colonic epithelium to an ultimately invasive cancer, as a result of pathologic transformation including molecular events in a variety of pathways [2]. Therefore, identification of critical factors that may be involved in initiation and progress of this multistep carcinogenic pathway may provide insights into effective therapies to combat CRC.

The SALL gene family, including four members (SALL1 to SALL4) was initially cloned based on DNA sequence homology to Drosophila gene spalt (sal) [3]. Sal is an essential homeotic gene for the development of the fly [4]. The human SALL gene family is involved in normal development. Structural properties of human SALL consist of several C2H2 zinc finger domains that can bind DNA, and in some cases, RNA and proteins [5]. In embryonic stem cells (ESCs), SALL4 has significant roles in the maintenance of pluripotency and self-renewal, efficient proliferation/stabilization, and cell fate decision [6,7]. It is also engaged in maintenance of human adult stem cell features [8]. Depending on ESCs context, the transcription factor SALL4 activates or represses various engrossed transcriptional networks involved in self-renewal and pluripotency by regulating crucial transcription factors and epigenetic modulators. SALL4 is also engaged in the regulation of chromatin remodeling by bridging transcriptional regulation and epigenetic regulation in stem cells [9]. Having direct interaction with key cell-signaling pathways such as Wnt and TGF-beta, SALL4 can play essential roles in cell fate decision and survival of ESCs [10,11]. SALL4 is an important regulator of the stemness state and survival, not only in several types of normal stem cells, but also in cancer cells and possibly cancer stem cells [12].

In adults, SALL4 expression is normally restricted to CD34+ hematopoietic stem/progenitor cells [10], and in adult mice, SALL4 is also predominantly expressed in testes and ovaries [13]. Nonetheless, SALL4 expression is reported in numerous malignancies, such as breast and lung cancers [14,15], precursor B-cell lymphoblastic lymphoma [16], myelodysplastic syndromes (MDS) [11], acute myeloid leukemia (AML) [10,11], endometriotic samples [17], ovarian germ cell tumors [18], all types of testicular germ cell tumors (GCTs) [19], all metastatic seminomas/dysgerminomas and embryonal carcinomas [20], and primary mediastinal yolk sac tumors (YSTs) [21]. It is suggested that SALL4 not only might be important in pathogenesis of GCTs, especially to maintain their poorly differentiated status, but also can be used as a highly specific marker to confirm the germ cell origin of a metastatic tumor, due to its sensitivity and specificity [19,20]. Furthermore, it is proposed that SALL4 may have diagnostic and therapeutic value in breast and lung cancers [14,15].

An intricate network of genetic and epigenetic aberrations involving various signaling pathways is responsible for the development of CRC. Unlike blood malignancies and GSTs, few studies have focused on the molecular epidemiology of SALL4 in other cancers and there are no reports of SALL4 gene expression and its probable role in CRC to date. Having considered its involvement in tumorigenesis, progression, and aggressiveness of various tumors, our aim in this study was to analyze SALL4 expression and its impact on clinicopathological features in CRC.

## Methods

### Tissue samples

Fresh tumoral and distant tumor-free colorectal tissues were obtained through colorectal surgery at Omid Oncology Hospital of Mashhad University of Medical Sciences (MUMS), Iran, from 46 patients who had not received any other therapeutic intervention such as chemo- or radiotherapy. After collection, the specimens were immediately treated with RNAlater solution (Qiagen, Hilden, Germany) and stored at −20°C until extraction. The ethics committee of MUMS approved the study and all patients formally declared their consent to be enrolled. Histopathological characteristics of tumor samples such as tumor size, location, and differentiation grading were recorded. Tumor-free colorectal tissues were also confirmed histologically to ensure that all the included samples are normal. Furthermore, based on the Union International Cancer TNM classification guidelines, the surgical stages of the tumoral samples were defined [22].

### cDNA synthesis and quantitative real-time-PCR

RNA extractions and cDNA syntheses were performed as described previously [23]. Quantitative real-time PCR was carried out using SYBR green PCR Master Mix (Fermentas, Lithuania), containing ROX as a reference dye on a Stratagene Mx-3000P real-time thermocycler (Stratagene, La Jolla, CA) with the primers presented in Table 1. The thermal profile included 10 min at 95°C followed by 40 cycles of 15 sec at 95°C, 30 sec at 57°C, and 45 sec at 72°C. Data were normalized to glyceraldehyde 3-phosphate dehydrogenase (GAPDH) expression [24] applying the comparative threshold cycle method. The PCR efficiencies for GAPDH and SALL4 were verified by generating related standard curves. The relative levels of SALL4 gene expression were compared based on fluorescence intensity changes of samples from tumor vs. corresponding normal tissues. A more than two-fold increase in expression was considered to be overexpression, while a more than two-fold decrease was considered to be underexpression. The range between those two values was interpreted as no change or normal expression. All experiments were performed in triplicate.

**Table 1:** **Primer sequences used for quantitative real**-**time RT**-**PCR**

	Forward primer sequence	Reverse primer sequence
SALL4	CCAAAGGCAACTTAAAGGTTCAC	CCGTGAAGACCAATGAGATCTC
GAPDH	GGAAGGTGAAGGTCGGAGTCA	GTCATTGATGGCAACAATATCCACT

### Statistical analysis

Data was analyzed using the SPSS 19.9 statistical package (SPSS, Chicago, IL). Based on requirements, either the χ^2^ or Fisher exacts test were applied to assess the correlations between gene expression and various histopathological features. Independent sample t test and ANOVA were used also to correlate gene expression levels and different categorical data. P values < 0.05 were considered to be statistically significant.

## Results

Fresh frozen colorectal tumors and corresponding normal-margin specimens of 46 newly-diagnosed patients were obtained through surgery prior to any other treatments, ensuring that histopathological characteristics of CRC tissues were not affected by therapeutic intervention. Based on microscopy, all the tumoral samples contained more than 75% tumor cells, with rare or no infiltrating cells. This procedure helped to ensure precise evaluation of gene expression in tumor cells compared to normal. SALL4 expression was analyzed by reverse transcription and real time-PCR amplification. The mean age ± standard deviation (SD) of the enrolled patients was 53.80 ± 14.89 (age range 21–86). The male-to-female ratio was 1.3:1 (26:20). The tumors were resected from distal and proximal regions of the colon with a size range from 3 to 10 cm (mean ± SD: 4.85 ± 1.55). Clinicopathological features of the patients are presented in Table 2.

**Table 2:** Clinicopathological features of the patients and correlation of SALL4 gene expression with them

**Factor**	**Number** (%)	**SALL4**		**P value**
		**↑**	**↓ / -**	
**Tumor invasion**				
**T1**, **T2**	8 (17.4%)	8	0	0.538
**T3**, **T4**	38 (82.6%)	32	6	
**Lymph node metastasis**				
**N0**	33 (71.7%)	28	5	**0**.047*
**N1**	9 (19.6%)	9	0	
**N2**	4 (8.7)	3	1	
**Stage**				
**I**/**II**	34 (73.9%)	29	5	0.397
**III**/**IV**	12 (26.1%)	11	1	
**Grade**				
**WD**	29 (63%)	25	4	**0**.020*
**MD**	16 (34.8%)	15	1	
**PD**	1 (2.2%)	0	1	
**Tumor location**				
**Proximal**	13 (28.3%)	11	2	0.611
**Distal**	33 (71.7%)	29	4	
**Sex**				
**Male**	26 (56.5%)	22	4	**0**.030*
**Female**	20 (43.5%)	18	2	

WD: Well differentiated; MD: Moderately differentiated; PD: Poorly differentiated.

*Significant correlation between SALL4 mRNA expression and clinicopathological feature.

### Upregulation of SALL4 in CRC

**Figure 1:**
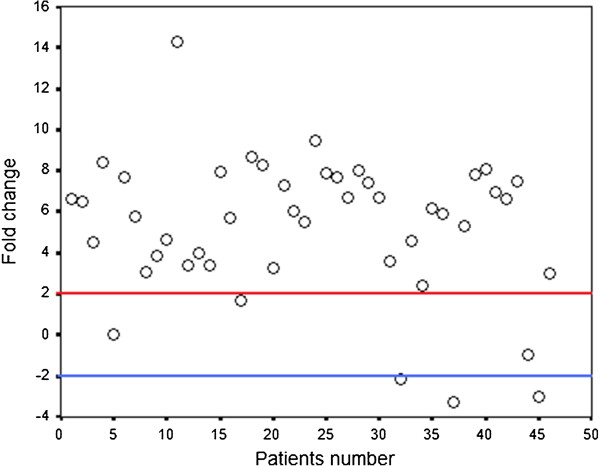
**Scatter plot representative of descriptive analysis of relative gene expression distribution of SALL4 in patients with CRC.** The Y axis indicates the relative gene expression, and the X axis represents the patients. Relative mRNA expression of more than two-fold in tumor tissues is considered as overexpression, less than minus two-fold as underexpression, and the range in-between is defined as normal.

**Figure 2:**
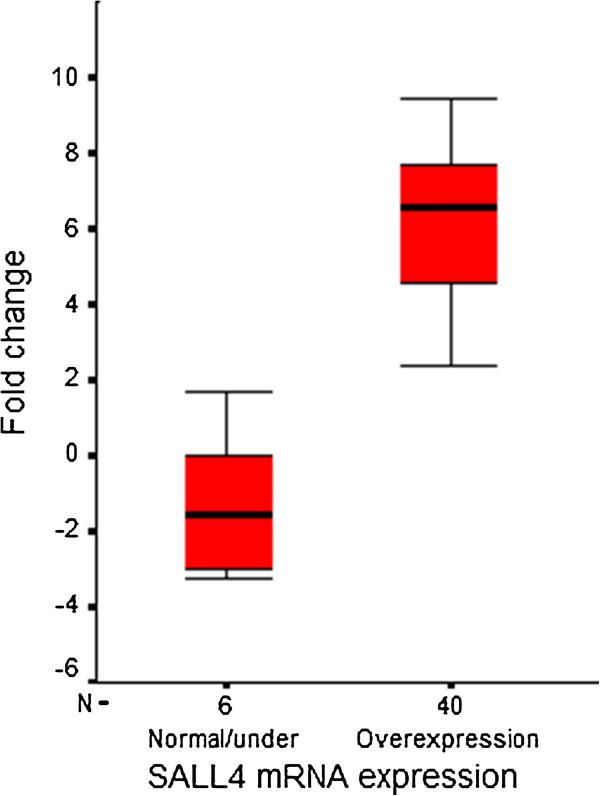
**Box plot representative of relative mRNA expression of SALL4 in CRC patients.** Quantitative analysis of SALL4 is represented as box plots. The Y axis indicates the fold change of relative mRNA expression, and the X axis represents patient groups. Box plots represent the lowest, lower quartile, median, upper quartile, and highest observations of fold changes in patients either with normal/underexpressed or overexpressed SALL4.

We compared SALL4 mRNA expression in 46 tumor specimens to their paired non-neoplastic colorectal epithelium by quantitative real time-PCR. The expression pattern of the gene in patients is represented as a scatter plot in Figure 1. Significant overexpression of SALL4 mRNA was detected in 40 of 46 of tumor specimens (87%, P < 0.0001). The minimum and maximum mRNA expression changes were −5.28- and 14.30-fold, respectively (Mean ± SD, 5.33 ± 3.59). Since a few patients showed normal or underexpression of the gene (six patients) we merged them to a single group in comparison with SALL4 overexpressed patients. The related means and standard deviations were 6.37±2.36 in patients with SALL4 overexpression and -1.61 ± 2.42 in other patients. Figure 2 schematically compares expression levels in both groups. Clinicopathological features of the patients with normal or underexpression of SALL4 mRNA are summarized in Table 3.

**Table 3:** Clinicopathological features of six tumor samples with normal or underexpression of SALL4 mRNA

**Clinicopathological feature**	**Description**
Sex	4 female, 2 male
Age	One patient is 21, others ranged 54-66
Location of tumor	2 proximal, 4 distal
Grade of tumor	4 WD, 1 MD, 1 PD
Stage of tumor	5 in stage II, 1 in stage III
Lymph node metastasis	5 without metastasis, 1 with metastasis
Depth of tumor invasion	All are invaded to adventitia (T3)
Size of tumor	Ranged 3–10 cm

WD: Well differentiated; MD: Moderately differentiated; PD: Poorly differentiated.

### Association of SALL4 expression with clinicopathological variables

To evaluate the clinicopathological consequences of SALL4 expression in CRC, we analyzed the correlation of various clinicopathological variables with SALL4 mRNA level (Table 2). We observed that SALL4 expression correlated with multiple indices of poor prognosis. SALL4 expression was significantly associated with tumor cell metastasis to lymph nodes (P < 0.05). Of nine patients with lymph node metastasis, all (100%) overexpressed SALL4 (mean ± SD: 6.45 ± 1.38), while in 15.2% of patients (5 of 33) without metastasis to lymph nodes SALL4 was not overexpressed (mean ± SD: 4.68 ± 3.72). Additionally, expression of SALL4 was associated with the grade of tumor cell differentiation. Twenty-five of 29 (86.2%) well-differentiated and fifteen of 16 (93.8%) moderately-differentiated tumors overexpressed SALL4 (P < 0.05). No significant associations were observed between SALL4 expression and other clinicopathological variables such as tumor stages or tumor invasion depth.

For further analyses, we assessed the correlations between SALL4 gene expression and different clinicopathological features in samples that overexpressed SALL4. SALL4 overexpression was significantly correlated with sex (P = 0.030, correlation coefficient: 0.384). SALL4 gene expression was significantly higher in males than females (mean ± SD: 5.25 ± 3.60 and 3.96 ± 2.45, respectively). Furthermore, in the same group, SALL4 expression was inversely correlated with patients’ ages (P = 0.034, correlation coefficient: -0.395). Thus, SALL4 expression is lower in tumor cells of older patients than younger ones.

In advanced tumor stages (stages III and IV), a significant inverse correlation was observed between SALL4 overexpression and the number of involved lymph nodes (P = 0.006, correlation coefficient: -0.737). In addition, in tumor samples without invasion to adventitia (T1, T2), SALL4 gene expression significantly correlated with patients’ ages (P = 0.033, correlation coefficient: -0.967). In such tumor samples, SALL4 expression was significantly higher in younger than older patients.

## Discussion

Expression analysis of SALL4 in normal and tumor colorectal tissues elucidated overexpression of SALL4 in a nearly 90% of CRC samples, where SALL4 expression was significantly associated with tumor cell metastasis to lymph nodes and the grade of tumor cell differentiation. There are some similar studies reporting the overexpression of SALL4 in different malignancies. In breast cancer, it is demonstrated that 86.1% of tumor samples showed elevated levels of SALL4 mRNA expression, where the increased levels of SALL4 mRNA expression were observed even in the early stages of tumors [14]. However, there was not any significant correlation between clinicopathological features of the patients and SALL4 mRNA expression in breast cancer [14]. Furthermore, it has been shown that 93% of lung tumors revealed SALL4 overexpression in mRNA levels [15]. These data may emphasize the role of SALL4 in carcinogenesis of different cancers specially by considering the results of related complementary studies.

Silencing of SALL4 in breast cancer cell line, MCF7, inhibits its propagation [14]. Furthermore, having silenced the SALL4 in SBC-1 lung cancer cells, Kobayashi et al. demonstrated that SALL4 has a critical role in the proliferation of lung cancer cells [15]. They showed that decreasing of SALL4 mRNA level to the baseline by 43% resulted in tragedic growth inhibition by 93.5% [15]. Therefore, SALL4 can be detected with high sensitivity and specificity and may have diagnostic and therapeutic value in breast and lung cancers [14,15].

It has been shown that all types of testicular GCTs express SALL4 protein in correlation with degree of tumor differentiation and suggested that SALL4 is essetial for the maintanance of poorly differentiated status [19]. Also, expression of SALL4 was detected in more than 90% of tumor cells of metastatic seminomas, dysgerminomas and embryonal carcinomas suggesting that SALL4 play a role in development of germ cell tumors [20]. Based on these results, SALL4 is introduced as a novel sensitive and specific marker for metastatic germ cell tumors and as a novel diagnostic marker for metastatic yolk sac tumors from the testis, ovary, and extragonadal sites [20].

In this study, six samples showed normal or underexpression of SALL4 mRNA. As summarized in Table 3, five of them had not metastasis to lymph nodes being in stage II and all of six samples showed tumor invasion to adventitia (T3), intrestingly. Since SALL4 mRNA expression is significantly correlated with metastasis of tumor cell to lymph node in CRCs, it may be remarkable that 83.3% of samples without SALL4 overexpression (5 of 6) have not showed metastasis to lymph node. These data may confirm the significant role of SALL4 mRNA expression in progression and lymph node metastasis of CRCs. We conclude from this results that increased expression of this stem cell transcription factor in CRC may be contributed in CRC tumorigenesis. Nonetheless, Analysis of different aspects of SALL4 in stem cell biology along with existing related data on CRC may help us to understand how SALL4 functions in CRC cells, and which cellular processes involving SALL4 may be involved in CRC pathogenesis.

SALL4, a member of the Spalt family, is a homeotic gene originally identified in Drosophila as a transcription factor required for development [25]. The transcriptional network of SALL4/OCT4/Nanog is crucial for the maintenance of “stemness” of ES cells [12] whereas SALL4, as a core factor, plays a dominant role in this regulatory network [8]. It activates OCT4 as a transcriptional factor and interacts with Nanog in a protein-protein complex [26,27]. Due to the same genomic binding sites of SALL4 and Nanog, reciprocal regulation of these proteins is suggested in governing pluripotency and self-renewal of ES cells [27]. Interestingly, overexpression of OCT4 and Nanog has been shown in CRC samples. Expression of OCT4 was increased with CRC stage progression [28]. Furthermore, overexpression of Nanog was significantly correlated with poor prognosis, lymph node metastasis, and TNM classification of colorectal cancer [29,30]. These data, along with SALL4 overexpression, which is reported here, lead us to hypothesize the existence of activated similar regulatory transcriptional networks in colorectal tumor cells playing essential roles in proliferation and self-renewal characteristics of tumor cells. Considering the existence of the stemness regulatory network of SALL4/OCT4/Nanog in CRC cells, we suggest that the pathogenesis of CRC and evolution of colorectal malignancy might be related to abundance and contribution of stem cell-like cells in the tumors. Our results support the hypothesis of cancer stem cell-based tumor development and progression.

SALL4 increases levels of histones H3–K4 and H3–K79 trimethylations in the Bmi-1 promoter in association with a methyltransferase in vivo [31]. Bmi-1 is a component of a polycomb group (PcG) multiprotein complex required to maintain the transcriptionally repressive state of many genes, including Hox genes, throughout development [32]. Our data shows SALL4 overexpression in significant correlation with lymph node metastasis and epithelial-mesenchymal transition (EMT). Furthermore, Bmi1 is overexpressed in CRC samples associated with degree of differentiation, status of lymph node metastasis, and TNM staging in colorectal cancer [33]. Combining these data and results, we propose that the role of SALL4 in lymph node metastasis may be due to its role in epigenetic modulation of Bmi-1, leading to repression of the involved genes in epithelial cell differentiation. Such sustained repression may cause EMT in CRC cells leading to the loss of cell adhesion and subsequent release of tumoral cells from the epithelium into the local lymph nodes. This may be an oncogenic role of SALL4 in cell fate decision in CRC through Bmi-1 activation. In support of this hypothesis, previous analysis of the SALL4/Bmi-1 regulatory pathway in cancer stem cells revealed the potential of this pathway as an attractive target for therapeutic intervention [34].

SALL4 is an important regulator not only of the stemness state and survival of several types of normal stem cells, but also the survival and expansion of cancer cells and possibly cancer stem cells [12]. SALL4 not only binds genes involved in important cell growth pathways such as Wnt, apoptosis, PTEN, and NF-kappaB signaling, but gene expression levels in these pathways are also affected by reduction of SALL4.

SALL4 regulates cell survival by targeting a wide range of genes in both pro- and anti-apoptotic pathways [35]. Furthermore, expression of such genes is regulated by SALL4 in ES cells [12]. In CRC samples, overexpression of SALL4 may have a fundamental role in tumor cell survival by transcriptional repression of pro-apoptotic genes such as PTEN. Interestingly, PTEN expression is decreased or absent within colorectal tumor cells compared to related normal tissues [36]. The lack of PTEN expression in CRC samples was encountered as a result of various mutations in the PTEN gene [36]. Based on our results, we also hypothesize a probable role for SALL4 overexpression in repression of PTEN transcription, which leads to down-regulation of PTEN in CRC cells. This hypothesis is supported by the following: first, down-regulation of SALL4 results in apoptosis [34], which may partly being due to activation of pro-apoptotic genes such as PTEN. It has been reported that SALL4 functions as a regulator of cell survival in human acute promyelocytic leukemia cells by targeting and regulating a wide range of genes in both pro-apoptotic and anti-apoptotic pathways [34]. Second, it has been established that PTEN has tumor-suppressive effects in CRC cells [37]. Therefore, SALL4 overexpression may regulate CRC cells survival by inhibiting apoptosis via repressing transcription of the pro-apoptotic and tumor suppressor gene PTEN.

## Conclusion

This study elucidates the clinical importance of stem cell marker SALL4 expression at the mRNA level for the first time in CRC. We have shown SALL4 may be a new molecular marker of metastatic tumor. Its probable role in CRC progression is confirmed by its overexpression in CRC and its correlation to lymph node metastasis, a determinant of poor prognosis. Overexpression of SALL4 mRNA in colorectal cancer may have a role in the progression of the disease and may shed a light in the elucidation of pathophsiology of colorectal cancer.

## Competing interests

The authors declare that they have no competing interests.

## Authors’ contributions

MMF designed the concept and conducted the experiments, analyzed data, drafted and edited the manuscript. MM and RR performed the majority of the work presented in this manuscript. AT was involved in surgery and tissue preparation. AJM was involved in drafting and edition. SB-N pathologically examined the samples. MRA had a critical scientific revision on the manuscript. All of the authors read and approved the final manuscript.
